# Predicting low muscle strength from routine fitness-monitoring data in hypertensive older adults living at high altitude: development and internal validation of interpretable models

**DOI:** 10.3389/fpubh.2026.1916266

**Published:** 2026-09-15

**Authors:** Ting Chen, Xin Zhao, Ruizhe Sun, Ba Bian, Weibao Liang

**Affiliations:** 1 Key Laboratory of Plateau Hypoxia Environment and Life and Health, School of Physical Education, Plateau Sports ad Health Research Center, Xizang Minzu University, Xianyang, China; 2 Tibet University for Nationalities Affiliated Hospital, Xianyang, China; 3 Tibet Autonomous Region Institute of Sports Science, Lhasa, China; 4 Medical College, Tibet University, Lhasa, China; 5 Faculty of Health Sciences and Sports, Macao Polytechnic University, Macao, China

**Keywords:** clinical prediction model, handgrip strength, high-altitude residence, hypertension, low muscle strength, machine learning, older adults

## Abstract

**Introduction:**

Low muscle strength based on grip-strength thresholds is a readily measurable marker of impaired muscle function, yet it is under-recognized among hypertensive older adults at high altitude. We developed a concurrent clinical prediction model using routine fitness-monitoring data and tested whether the full feature set added information beyond sex, age, and site/altitude.

**Methods:**

This cross-sectional secondary analysis included 433 hypertensive adults aged 60–69 years from three sites in the National Physical Fitness Monitoring program in the Tibet Autonomous Region. Low muscle strength was defined using Asian Working Group for Sarcopenia 2019 grip-strength thresholds (men <28 kg; women <18 kg). Fifteen predictors were prespecified for routine availability and plausible relevance to body composition, cardiometabolic status, respiratory or neuromotor function, physical activity, and site/altitude. Elastic-net logistic regression, random forest, and histogram gradient boosting were evaluated by repeated nested cross-validation. *Post hoc* analyses included simple benchmark models, a sex-excluded model, sex-stratified models, threshold operating characteristics, and leave-one-site/altitude-band-out internal–external validation.

**Results:**

Low muscle strength was present in 171/433 participants (39.5%). Random forest had the highest AUC point estimate (0.788, 95% CI 0.742–0.831), but its interval overlapped with elastic-net logistic regression (0.781, 0.732–0.825) and histogram gradient boosting (0.774, 0.729–0.819). Elastic-net was designated as the candidate model because calibration was closest to ideal (slope 0.99, 95% CI 0.78–1.24; intercept −0.00, −0.22 to 0.24) and it provides a transportable equation. Its PR-AUC was 0.725 versus a no-skill baseline of 0.395. Incremental discrimination over sex plus age plus site/altitude was small (ΔAUC 0.016, 95% CI − 0.009 to 0.041). AUC was 0.705 without sex, 0.653 under a sex-specific tertile outcome, and 0.626–0.759 when each site was held out.

**Conclusion:**

Routine fitness-monitoring variables contain information about concurrent low muscle strength, but much of the apparent performance is explained by the sex-specific outcome definition and site/altitude structure. Direct grip-strength measurement remains preferable whenever feasible. The elastic-net model should be regarded only as a candidate for opportunistic triage when a valid grip measurement is unavailable or unusable; independent external validation, recalibration, and prospective impact evaluation are required before implementation.

## Introduction

Hypertension is among the leading modifiable contributors to cardiovascular disease and premature death worldwide, and its prevalence rises steeply with age (1). Although awareness, treatment and control have improved in some settings, they remain inadequate in many older and rural populations, leaving a large share of older adults exposed to the long-term consequences of elevated blood pressure (2).

People living permanently at high altitude face an additional cardiovascular challenge. Sustained hypobaric hypoxia elicits sympathetic activation, endothelial dysfunction and vasoconstriction, and a higher prevalence of hypertension has been reported with increasing altitude in Tibetan communities (3). High-altitude residence also entails adaptations in oxygen transport, vascular regulation and tissue metabolism that may influence functional aging; however, environmental effects are difficult to separate from site-specific differences in lifestyle, nutrition and socioeconomic conditions (4, 5).

Aging is accompanied by progressive loss of muscle mass and function, which strongly predicts falls, physical disability, hospitalization and death (6–8). Low muscle strength is increasingly viewed as the central and most readily measurable component of this syndrome. Consensus definitions from the Asian Working Group for Sarcopenia (AWGS 2019) and the European Working Group on Sarcopenia in Older People (EWGSOP2) place handgrip strength at the core of case finding and use sex-specific thresholds (9, 10). Grip strength is inexpensive, reproducible and prognostically informative across diverse populations (11, 12). Because a complete sarcopenia diagnosis additionally requires assessment of muscle mass and physical performance, an outcome based on grip strength alone is most accurately described as low muscle strength based on grip-strength thresholds, not sarcopenia.

Hypertension and impaired muscle health may share vascular and metabolic pathways, including arterial stiffness, microvascular dysfunction, oxidative stress, chronic low-grade inflammation and insulin resistance (13). Chronic hypoxia may add mitochondrial and metabolic remodeling, altered proteostasis and catabolic signaling in skeletal muscle (14, 15). Tibetan data also suggest that muscle mass, performance and locally derived grip-strength reference values vary across high-altitude settings, although these cross-sectional patterns cannot isolate hypoxia from other site-level determinants (16). These mechanisms make a high-altitude hypertensive population biologically relevant, but they do not justify causal interpretation of site/altitude associations.

National physical-fitness monitoring programs capture multidimensional information on anthropometry, body composition, cardiovascular status, respiratory and neuromotor function, and habitual activity. A concurrent prediction model can combine these already recorded variables to flag records requiring confirmatory assessment. Its added value is necessarily limited: when a valid grip-strength measurement can be obtained during the same encounter, direct dynamometry is simpler and should remain the preferred assessment. The potential use considered here is therefore opportunistic triage of existing monitoring records when grip strength was omitted, invalid or unavailable, rather than replacement of grip testing or diagnosis of full sarcopenia.

We therefore developed and internally validated interpretable models for concurrent prediction of low muscle strength based on AWGS 2019 grip-strength thresholds among hypertensive adults aged 60–69 years at three high-altitude sites. We compared elastic-net logistic regression with two tree-ensemble methods, quantified incremental performance over simple sex-, age- and site/altitude-based benchmarks, examined performance without sex and within sex strata, and conducted leave-one-site/altitude-band-out internal–external validation. The intended output was a fully specified candidate model for future external validation, not an implementation-ready screening instrument.

## Materials and methods

### Study design and reporting

This was a cross-sectional secondary analysis framed as model development with internal validation. The core analysis plan—outcome definition, candidate predictors, model families, repeated nested cross-validation, primary performance measures, and sensitivity analyses—was specified before model fitting but was not prospectively registered. Benchmark models, sex-excluded and sex-stratified analyses, threshold-specific operating characteristics, and leave-one-site/altitude-band-out internal–external validation were *post-hoc* analyses undertaken to examine robustness, structural dependence, and transportability. Reporting follows TRIPOD+AI (17) and STROBE (18); completed checklists are provided as Supplementary files. Detailed computational methods, complete model coefficients, preprocessing constants, software versions and random seeds are reported in Supplementary material 1, and executable code and machine-readable specifications are supplied in Supplementary material 2. The study was approved by the Biomedical Ethics Committee of Xizang Minzu University (Approval No. 2021–31), and all participants provided written informed consent.

### Setting and participants

Data were drawn from the de-identified analytic file of the Fifth National Physical Fitness Monitoring (NPFM) program in the Tibet Autonomous Region. The national Fifth NPFM fieldwork window was August–December 2020, and the Lhasa component was formally launched on 1 September 2020; participant-level dates and the site-specific completion dates for Nyingchi and Nagqu were not retained (19, 20). The file covered Nyingchi (Linzhi; approximately 3,100 m), Lhasa (approximately 3,650 m), and Nagqu (approximately 4,500 m). Because each altitude value corresponded to one site, altitude and site were perfectly collinear; throughout this report, the variable is therefore described as the site/altitude band rather than an independent environmental exposure.

Eligible participants were community-dwelling permanent residents aged 60–69 years who had lived locally for at least 1 year. The monitoring algorithm classified hypertension when the mean of three seated blood-pressure readings was SBP ≥ 140 mmHg and/or DBP ≥ 90 mmHg, or when a participant reported physician-diagnosed hypertension with current antihypertensive treatment. Every participant in the available analytic file also met the measured-BP criterion; the cohort was therefore effectively selected by BP measured at the monitoring visit. Phenotypes were defined from the recorded values as isolated systolic hypertension (SBP ≥ 140 and DBP < 90 mmHg), isolated diastolic hypertension (SBP < 140 and DBP ≥ 90 mmHg), or combined hypertension (SBP ≥ 140 and DBP ≥ 90 mmHg). The extract did not retain medication class, regimen, adherence, or treatment-control status. Original monitoring exclusions included secondary hypertension, severe cognitive impairment or communication barriers precluding questionnaire completion, and physical disability preventing grip-strength testing or routine activity assessment. Of 454 eligible records, 21 lacking grip strength were excluded, leaving 433 participants (Figure 1).

**Figure 1:**
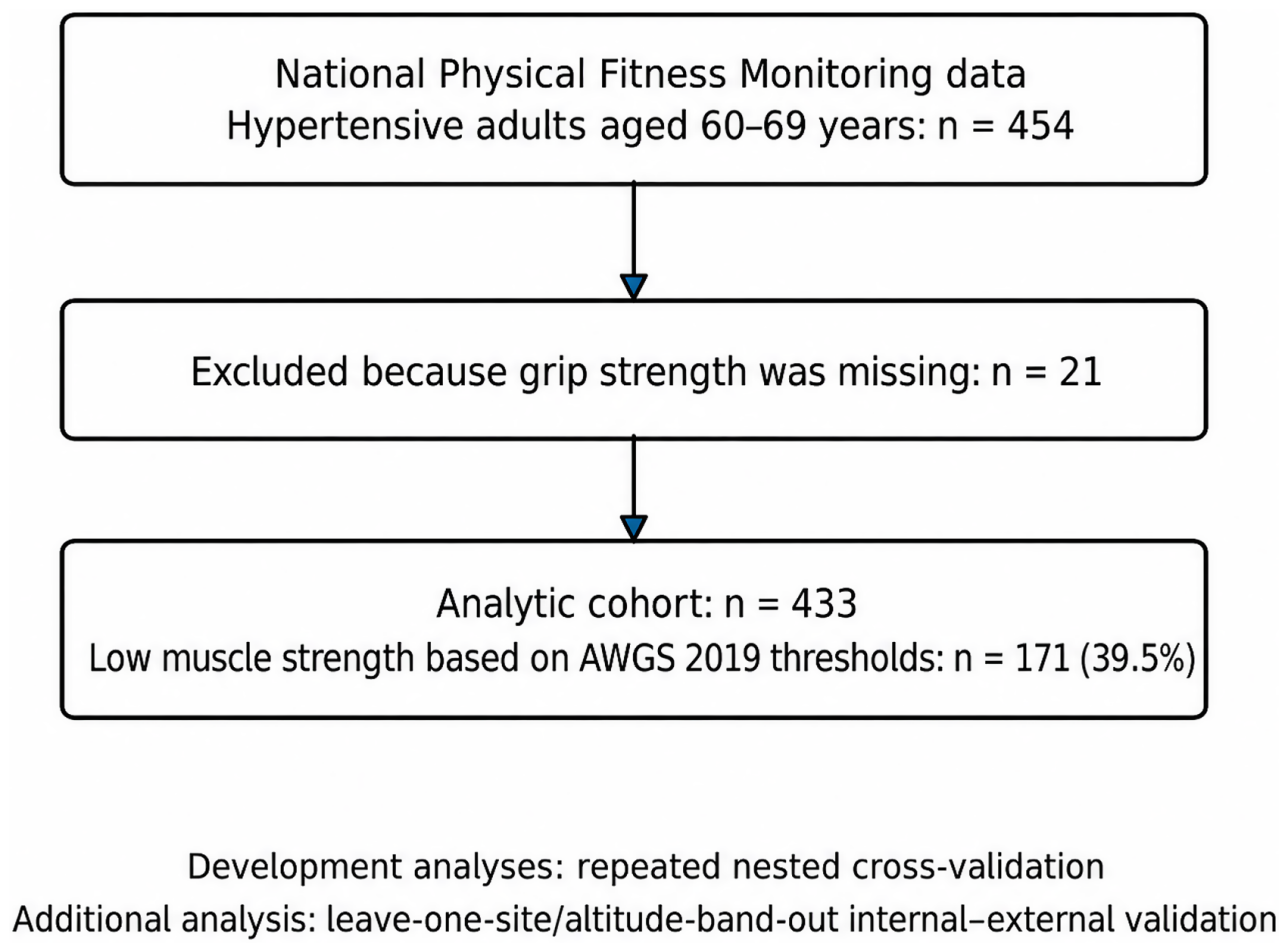
Participant flow and analysis overview. Of 454 hypertensive adults aged 60–69 years at three site/altitude bands, 21 were excluded because grip strength was missing, leaving 433 participants. Low muscle strength was defined using AWGS 2019 sex-specific grip-strength thresholds. Development used repeated nested cross-validation; internal–external evaluation used leave-one-site/altitude-band-out internal–external validation.

### Measurement procedures

Age and sex were obtained by face-to-face interview. Field staff were trained in instrument operation and standardized testing, and the Fifth NPFM used automated data upload and real-time quality control (19–21). Blood pressure was measured on the right upper arm after at least 5 min of seated rest using either a calibrated mercury sphygmomanometer or a validated automated upper-arm device, with an upper-arm cuff selected according to the field protocol. Three consecutive readings were obtained at 1–2-min intervals, and their arithmetic mean was recorded as an integer value in mmHg. Participant-level device identifiers, cuff dimensions, and the three individual readings were not retained. Recorded BP displayed site-varying terminal-digit heaping, particularly at the 3,100-m site; model robustness was therefore also assessed after excluding SBP and DBP.

Grip strength was assessed with a digital handgrip dynamometer (WCS-100, China). The grip span was adjusted to a comfortable position; participants stood upright with the feet naturally apart and the tested arm hanging alongside the body, without allowing the dynamometer to contact the body. Two maximal trials were performed with the dominant hand, and the higher value in kilograms was retained (21).

Height and weight were measured using standard NPFM instruments, and BMI was calculated as weight in kilograms divided by height in meters squared. Body fat percentage was recorded by bioelectrical impedance analysis; the analyzer make/model and pre-test hydration, meal, bladder, and exercise conditions were not retained. Twelve values outside 5–60% were treated as missing before modeling, and a sensitivity analysis excluding body fat was performed because measurement validity could not be independently verified. Vital capacity was measured with a spirometer after maximal inspiration followed by complete expiration without a second inspiration; two trials were performed and the maximum was retained in milliliters. Sit-and-reach, eyes-closed one-leg standing, and choice reaction time followed the NPFM older-adult procedures: two trials were performed and the best result was retained, and reaction time comprised responses to five audiovisual signals. Exact make/model identifiers for the body-composition analyzer, spirometer, and functional-test devices were unavailable in the extract (21).

### Outcome

The primary outcome was low muscle strength based on the AWGS 2019 sex-specific handgrip thresholds (men <28 kg; women <18 kg) (9). Because the thresholds differ by sex, sex is structurally linked to the binary outcome and was expected to be a strong predictor. Two alternative definitions were examined: EWGSOP2 thresholds (men <27 kg; women <16 kg) (10) and the lowest third of grip strength within each sex. The latter defined relative position within each sex and removed the different absolute male and female cut-offs, thereby reducing discrimination attributable solely to those absolute thresholds. Grip-strength distributions and AWGS 2019 cut-offs are shown in Supplementary Figure S1.

### Candidate predictors

Fifteen candidate predictors were selected without univariable outcome screening. Selection was based on routine availability and prespecified domains plausibly relevant to concurrent muscle function: age and sex; body composition (body mass index, body fat percentage, and waist-to-hip ratio); cardiovascular measures (resting heart rate, SBP, and DBP); respiratory and neuromotor or functional measures (vital capacity, sit-and-reach flexibility, one-leg standing balance with eyes closed, and choice reaction time); habitual exercise frequency and session duration; and site/altitude band. Grip strength was excluded because it defined the outcome. Raw walking days and minutes were retained for cohort description but were not candidate predictors. Exercise frequency was coded as sedentary, low frequency (1–2 times/week), or regular (≥3 times/week), and session duration as <30, 30–60, or ≥60 min. The primary models retained the source fields as recorded. Because session duration is not logically interpretable for a participant reporting no exercise, a sensitivity analysis reassigned the 21 sedentary records with a longer duration category to <30 min. Functional variables were retained as concurrent triage markers, not causal antecedents. Sources, units, and preprocessing are detailed in Supplementary Table S1; missingness is summarized in Supplementary Table S2; and pairwise correlations are shown in Supplementary Figure S2. Values judged physiologically implausible were set to missing before modeling (body fat <5% or >60%; waist-to-hip ratio >1.30).

### Missing data and sample size

The available sample size was fixed by the secondary dataset; no outcome-based prospective sample-size calculation was possible. There were 171 outcome events and 17 effective predictor parameters before penalization, giving approximately 10 events per parameter. Because this is limited for flexible models, complexity was constrained through penalization, shallow trees and a small prespecified feature set (22, 23). Missing values were imputed within each cross-validation training fold using the median for continuous predictors and the most frequent level for categorical predictors; the fitted imputation parameters were then applied to the corresponding validation fold. This prevented information leakage but, as a single deterministic strategy, did not propagate imputation uncertainty. A complete-case analysis was therefore performed as a sensitivity analysis (*n* = 325).

### Model development

Three model families were compared: elastic-net logistic regression, random forest (24), and histogram-based gradient boosting (25). For logistic regression, continuous predictors were median-imputed and standardized, and categorical predictors were mode-imputed and one-hot encoded with fixed reference levels; all preprocessing was estimated within training data only (26). Random forest and gradient boosting used the same fold-specific imputation and encoding but no scaling. Inner four-fold cross-validation selected hyperparameters by AUC from prespecified grids (Supplementary Table S3). Because the outcome prevalence was 39.5%, neither class weighting nor synthetic oversampling was used, thereby avoiding avoidable distortion of predicted probabilities. After comparison, elastic-net logistic regression was designated as the candidate model for future validation because it combined similar discrimination with the closest-to-ideal calibration and a directly transferable equation. The modal configuration across outer folds (C = 0.30; l1 ratio = 1.00) was refitted to all 433 participants solely to provide coefficients and preprocessing constants for external validation; performance was not estimated from this full-data fit.

### Model validation

Primary performance was estimated by repeated nested cross-validation: a stratified five-fold outer loop repeated twice and a four-fold inner loop for tuning. Each participant therefore received two out-of-fold probabilities, which were averaged before performance estimation. Discrimination was summarized by the area under the receiver-operating-characteristic curve (AUC) and area under the precision–recall curve (PR-AUC); the event prevalence (0.395) was reported as the no-skill PR-AUC baseline. Overall accuracy was summarized by the Brier score. Calibration was assessed by calibration-in-the-large (intercept), calibration slope and graphical calibration. Net benefit was evaluated by decision-curve analysis from threshold probabilities 0.05–0.60 (27–29). Participant-level bootstrap resampling (2000 replicates) provided 95% confidence intervals for primary performance measures. Precision–recall curves are shown in Supplementary Figure S3. Because model comparisons used the same out-of-fold participants, incremental AUC and Brier-score differences were estimated by paired bootstrap resampling (Supplementary Table S4); subgroup AUCs are shown in Supplementary Figure S4.

### Decision context and threshold analysis

A positive model flag was defined as a prompt for standardized confirmatory grip-strength measurement. If low strength were confirmed, subsequent assessment would include muscle mass and physical performance rather than treating the model output as a sarcopenia diagnosis. Because direct grip testing is low burden, thresholds of 0.20–0.40 were considered clinically plausible exploratory operating points; 0.50 and the Youden-index threshold were also reported. For each threshold, sensitivity, specificity, positive and negative predictive values, accuracy and net benefit were calculated from candidate-model out-of-fold probabilities (Supplementary Table S5). False positives would lead mainly to additional assessment and possible anxiety, whereas false negatives could delay confirmatory assessment and intervention.

### Benchmark, sex-specific and internal–external analyses

*Post hoc* benchmark models used sex alone, sex plus age, sex plus site/altitude, and sex plus age plus site/altitude. Their out-of-fold probabilities were generated on the same outer splits, and paired bootstrap differences quantified the incremental value of the full elastic-net feature set. We also fitted a full model without sex and separate models within men and women (Supplementary Table S4; Supplementary Figure S5). Sensitivity and subgroup analyses are reported in Supplementary Tables S6, S7. Internal–external validation left each site/altitude band out in turn: the model was tuned using the other two sites and evaluated once in the held-out site (Supplementary Table S8; Supplementary Figure S6). This analysis tests between-site transportability within the same cohort structure but does not constitute independent external validation (30, 31).

### Interpretation and candidate-model specification

Interpretation focused on prediction rather than causation. Random-forest permutation importance was used to rank variables descriptively, and partial-dependence plots summarized model-based patterns for the four leading continuous variables (25). A separate unpenalized multivariable logistic model provided mutually adjusted odds ratios for descriptive association, with continuous variables expressed per one standard deviation and 95% confidence intervals from 1,200 bootstrap samples. No simplified score was retained, to avoid presenting competing end products. The elastic-net logistic model is the sole candidate for future external validation; its full equation, coefficients, imputation values, standardization constants and categorical reference levels are reported in Supplementary Tables S9, S10 and machine-readable files.

### Software

Analyses were conducted in Python 3.13.5 with scikit-learn 1.8.0, NumPy 2.3.5 and SciPy 1.17.0 (26). Package versions, random seeds, grids and exported specifications are listed in Supplementary Table S11 and Supplementary material 2.

## Results

### Participant characteristics

The 433 participants were evenly distributed by sex (213 men and 220 women) and across the three site/altitude bands (153 at 3100 m, 111 at 3650 m and 169 at 4500 m). Low muscle strength based on AWGS thresholds was present in 171 participants (39.5%). Prevalence was 58.2% in men and 21.4% in women, a difference that partly reflects the sex-specific thresholds, and 30.1, 36.0 and 50.3% across the 3,100-, 3650- and 4,500-m sites, respectively. These site differences are descriptive and cannot be attributed to altitude independently. Hypertension phenotypes comprised 42 isolated systolic, 207 isolated diastolic and 184 combined cases. Baseline characteristics are shown in Table 1, and missingness is summarized in Supplementary Tables S1, S2.

**Table 1:** Baseline characteristics of the analytic sample by low-muscle-strength status.

Characteristic	Overall (*n* = 433)	Normal (*n* = 262)	Low muscle strength (*n* = 171)	*p* value	SMD
No. of participants	433	262	171		
Age, years	64.4 ± 2.8	64.1 ± 2.8	64.8 ± 2.8	0.008	−0.26
BMI, kg/m^2	25.3 ± 3.7	25.4 ± 3.9	25.2 ± 3.2	0.613	0.05
Body fat, %	28.8 ± 11.5	30.5 ± 10.4	26.1 ± 12.6	<0.001	0.38
Waist-to-hip ratio	0.9 ± 0.1	0.9 ± 0.1	1.0 ± 0.1	<0.001	−0.4
Waist circumference, cm	86.3 ± 11.2	85.6 ± 10.9	87.3 ± 11.6	0.144	−0.15
Hip circumference, cm	91.4 ± 10.3	91.9 ± 10.9	90.7 ± 9.2	0.224	0.12
Resting heart rate, beats/min	78.8 ± 9.0	79.3 ± 8.7	78.1 ± 9.4	0.207	0.13
Systolic BP, mmHg	137.1 ± 11.5	135.9 ± 12.0	138.8 ± 10.5	0.009	−0.25
Diastolic BP, mmHg	96.4 ± 8.7	95.9 ± 7.7	97.1 ± 9.9	0.182	−0.13
Pulse pressure, mmHg	40.7 ± 13.0	40.1 ± 12.8	41.7 ± 13.3	0.198	−0.13
Vital capacity, mL	1625.1 ± 488.0	1646.4 ± 487.3	1592.4 ± 488.8	0.303	0.11
Sit-and-reach, cm	2.5 ± 7.0	2.6 ± 6.6	2.4 ± 7.5	0.754	0.03
One-leg balance, s	8.2 ± 9.0	7.9 ± 7.4	8.6 ± 11.1	0.437	−0.08
Reaction time, s	0.8 ± 0.3	0.8 ± 0.2	0.9 ± 0.3	0.091	−0.18
Grip strength, kg	24.5 ± 7.6	27.8 ± 7.4	19.4 ± 4.7	<0.001	1.34
Walking, days/week	5.3 ± 1.7	5.3 ± 1.8	5.4 ± 1.6	0.669	−0.04
Walking, min/day	39.7 ± 41.2	35.1 ± 17.7	46.7 ± 61.2	0.024	−0.26
Sex, n (%)				<0.001	
Male	213 (49.2)	89 (34.0)	124 (72.5)		0.77
Female	220 (50.8)	173 (66.0)	47 (27.5)		
Site/altitude band, n (%)				<0.001	
3,100 m (Nyingchi)	153 (35.3)	107 (40.8)	46 (26.9)		−0.29
3,650 m (Lhasa)	111 (25.6)	71 (27.1)	40 (23.4)		
4,500 m (Nagqu)	169 (39.0)	84 (32.1)	85 (49.7)		
Hypertension phenotype, n (%)				0.157	
Isolated systolic	42 (9.7)	24 (9.2)	18 (10.5)		0.05
Isolated diastolic	207 (47.8)	135 (51.5)	72 (42.1)		
Combined	184 (42.5)	103 (39.3)	81 (47.4)		
Exercise frequency, n (%)				0.012	
Sedentary	380 (87.8)	220 (84.0)	160 (93.6)		0.3
Low frequency	24 (5.5)	19 (7.3)	5 (2.9)		
Regular	29 (6.7)	23 (8.8)	6 (3.5)		
Session duration, n (%)				<0.001	
<30 min	373 (86.1)	212 (80.9)	161 (94.2)		0.4
30–60 min	49 (11.3)	41 (15.6)	8 (4.7)		
≥60 min	11 (2.5)	9 (3.4)	2 (1.2)		

Data are mean ± SD or n (%). *p* values are from Welch’s t-test (continuous variables) or chi-square test (categorical variables); they are descriptive and were not used for predictor selection. SMD, standardized mean difference. Grip strength is shown descriptively and defines the outcome. Site and altitude are perfectly collinear. Hypertension phenotypes were defined as isolated systolic (SBP ≥140 and DBP <90 mmHg), isolated diastolic (SBP <140 and DBP ≥90 mmHg), or combined (SBP ≥140 and DBP ≥90 mmHg). Body fat percentage and waist-to-hip ratio are reported after cleaning implausible values. Session-duration categories are shown as recorded; a sensitivity analysis reassigned sedentary participants with longer duration categories to <30 min.

### Model performance

Random forest had the highest discrimination point estimate (AUC 0.788, 95% CI 0.742–0.831), followed by elastic-net logistic regression (0.781, 0.732–0.825) and histogram gradient boosting (0.774, 0.729–0.819), but the confidence intervals overlapped (Table 2; Figure 2A). PR-AUCs were 0.734, 0.725 and 0.722, respectively, all above the no-skill baseline of 0.395 (Supplementary Figure S3). The candidate elastic-net model had a Brier score of 0.181 (95% CI 0.162–0.200), calibration slope 0.99 (0.78–1.24) and calibration intercept −0.00 (−0.22 to 0.24). Calibration was less stable for the tree ensembles, particularly the random forest (slope 1.33, 1.06–1.64; Table 2; Figure 2B).

**Table 2:** Predictive performance of the three model families under repeated nested cross-validation.

Model	AUC (95% CI)	PR-AUC (95% CI)	Brier (95% CI)	Calibration slope (95% CI)	Calibration intercept (95% CI)
Elastic-net logistic (candidate)	**0.781 (0.732–0.825)**	**0.725 (0.656–0.797)**	**0.181 (0.162–0.200)**	**0.99 (0.78–1.24)**	**−0.00 (−0.22 to 0.24)**
Random forest	0.788 (0.742–0.831)	0.734 (0.665–0.796)	0.180 (0.165–0.197)	1.33 (1.06–1.64)	0.13 (−0.09 to 0.38)
Histogram gradient boosting	0.774 (0.729–0.819)	0.722 (0.650–0.785)	0.184 (0.164–0.204)	0.86 (0.69–1.07)	−0.04 (−0.27 to 0.20)

Metrics were calculated from participant-level averaged out-of-fold predictions. Values are estimates (95% CI) from 2000 participant-level bootstrap samples. The no-skill PR-AUC equals the event prevalence (0.395). Ideal calibration slope = 1 and intercept = 0. Confidence intervals overlap for AUC. Elastic-net logistic regression was selected as the candidate for external validation because it combined similar discrimination with calibration closest to ideal and a reproducible equation. AUC, area under the receiver-operating-characteristic curve; PR-AUC, area under the precision–recall curve. Bold values indicate the performance estimates for the elastic-net logistic regression model selected as the candidate for future external validation.

**Figure 2:**
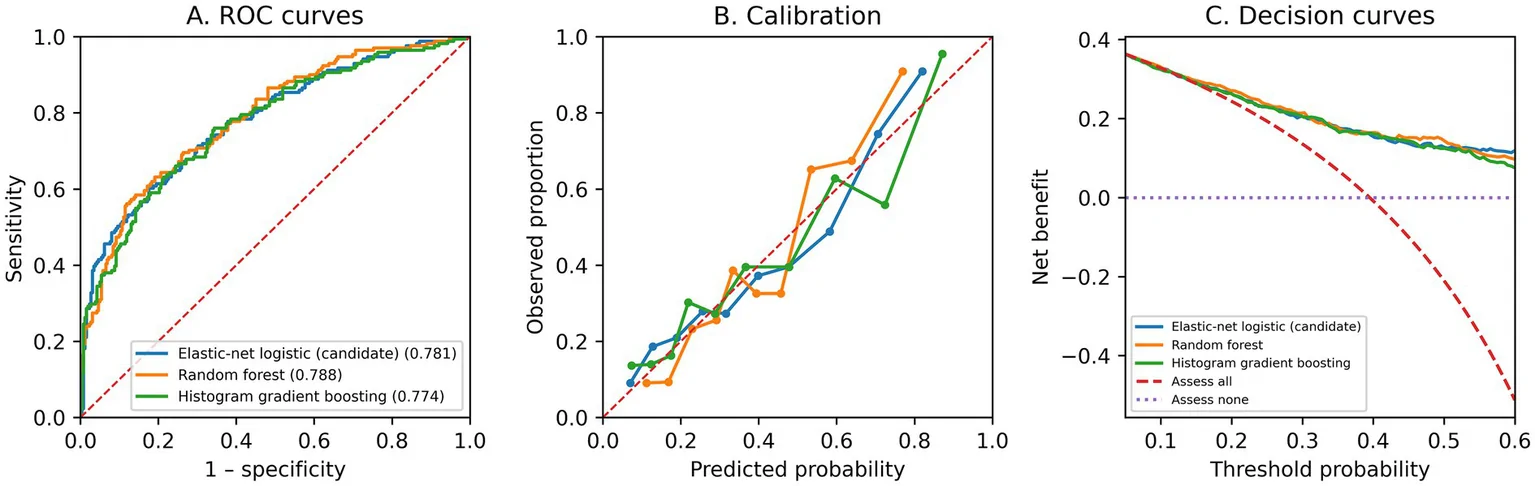
Predictive performance of the three model families. **(A)** Receiver-operating-characteristic curves. **(B)** Calibration plots using deciles of out-of-fold probability; the diagonal denotes perfect calibration. **(C)** Decision curves from threshold probabilities 0.05–0.60 relative to assess-all and assess-none strategies. Elastic-net logistic regression is the candidate model for future external validation. Estimates are based on participant-level averaged out-of-fold predictions from repeated nested cross-validation.

### Benchmark, sex-specific and internal–external performance

Simple benchmark performance showed that much of the signal was captured by structurally linked variables. AUC was 0.660 (95% CI 0.605–0.713) for sex alone, 0.737 (0.691–0.781) for sex plus age, 0.732 (0.681–0.783) for sex plus site/altitude, and 0.765 (0.718–0.811) for sex plus age plus site/altitude. Relative to the last benchmark, the full elastic-net candidate improved AUC by 0.016 (paired 95% CI − 0.009 to 0.041) and reduced the Brier score by 0.0067 (−0.0031 to 0.0164). Excluding sex reduced AUC to 0.705 (0.649–0.754). Separate sex-specific models yielded AUCs of 0.783 (0.721–0.844) in men and 0.640 (0.545–0.725) in women, with calibration slopes of 0.80 and 0.64 and wide intervals (Supplementary Table S4; Supplementary Figure S5).

In leave-one-site/altitude-band-out internal–external validation, AUC was 0.626 (95% CI 0.535–0.716) at 3100 m, 0.695 (0.583–0.800) at 3650 m and 0.759 (0.683–0.829) at 4500 m. Calibration varied materially: the corresponding intercepts were −0.57, −0.24 and 0.42, and slopes were 0.31, 0.74 and 1.15. Pooled held-out predictions had an AUC of 0.695 and calibration slope of 0.62 (Supplementary Table S8; Supplementary Figure S6), indicating between-site heterogeneity and a likely need for recalibration in new settings.

### Operating characteristics and decision curves

At exploratory candidate-model thresholds of 0.20, 0.30 and 0.40, sensitivity was 0.901, 0.784 and 0.684, respectively; specificity was 0.382, 0.595 and 0.710. At 0.30, positive and negative predictive values were 0.558 and 0.808. The Youden-index threshold was 0.481 (sensitivity 0.596; specificity 0.824), and at 0.50 sensitivity was 0.556 and specificity 0.844 (Supplementary Table S5). The candidate’s out-of-fold decision curve lay above assess-all and assess-none reference strategies through the clinically emphasized 0.20–0.40 range (Figure 2C). These are internally estimated operating characteristics, not deployment thresholds.

### Variable contributions and associations

In the descriptive multivariable logistic model (Table 3), male sex showed the largest adjusted association with low muscle strength (odds ratio [OR] 15.32, 95% CI 8.47–43.83), as expected from the outcome definition. Higher site/altitude band (OR per SD 2.61, 1.90–4.34), slower reaction time (1.77, 1.35–2.55), older age (1.42, 1.14–1.89), and higher recorded body fat percentage (1.46, 1.004–2.30) were also associated with higher odds, whereas a reported session duration of 30–60 min was associated with lower odds than <30 min (0.33, 0.08–0.81). The ≥60-min category was sparse (*n* = 11) and highly unstable. Random-forest permutation importance ranked sex first, followed by site/altitude, reaction time, body fat percentage, body mass index, and waist-to-hip ratio (Figure 3). Because body-fat device and pre-test metadata were unavailable, its association and importance are measurement-limited. These are concurrent, sample-specific associations and importance measures, not causal effects.

**Table 3:** Multivariable logistic-regression associations with low muscle strength.

Variable	Adjusted OR (95% CI)
Sex: male (vs female)	15.32 (8.47–43.83)*
Site/altitude band (per 1 SD)	2.61 (1.90–4.34)*
Reaction time (per 1 SD)	1.77 (1.35–2.55)*
Session: 30–60 min (vs < 30)	0.33 (0.08–0.81)*
Session: ≥60 min (vs < 30)	0.38 (<0.01–2.11)
Body fat % (per 1 SD)	1.46 (1.004–2.30)*
Age (per 1 SD)	1.42 (1.14–1.89)*
Diastolic BP (per 1 SD)	1.18 (0.89–1.57)
Vital capacity (per 1 SD)	0.82 (0.61–1.07)
One-leg balance (per 1 SD)	1.14 (0.91–1.47)
Exercise: regular (vs sedentary)	0.89 (0.19–3.10)
Waist-to-hip ratio (per 1 SD)	0.90 (0.63–1.29)
Resting heart rate (per 1 SD)	0.93 (0.71–1.21)
BMI (per 1 SD)	0.94 (0.69–1.22)
Systolic BP (per 1 SD)	1.06 (0.79–1.48)
Sit-and-reach (per 1 SD)	0.97 (0.71–1.31)
Exercise: low frequency (vs sedentary)	1.02 (0.14–5.43)

Odds ratios are from one descriptive multivariable logistic model. Continuous variables are expressed per 1-SD increase; categorical variables use the stated reference. Values are OR (95% CI) from 1,200 participant-level bootstrap samples. *CI excludes 1. The ≥60-min session category is sparse (*n* = 11) and its estimate is unstable. Associations are cross-sectional and should not be interpreted causally.

**Figure 3:**
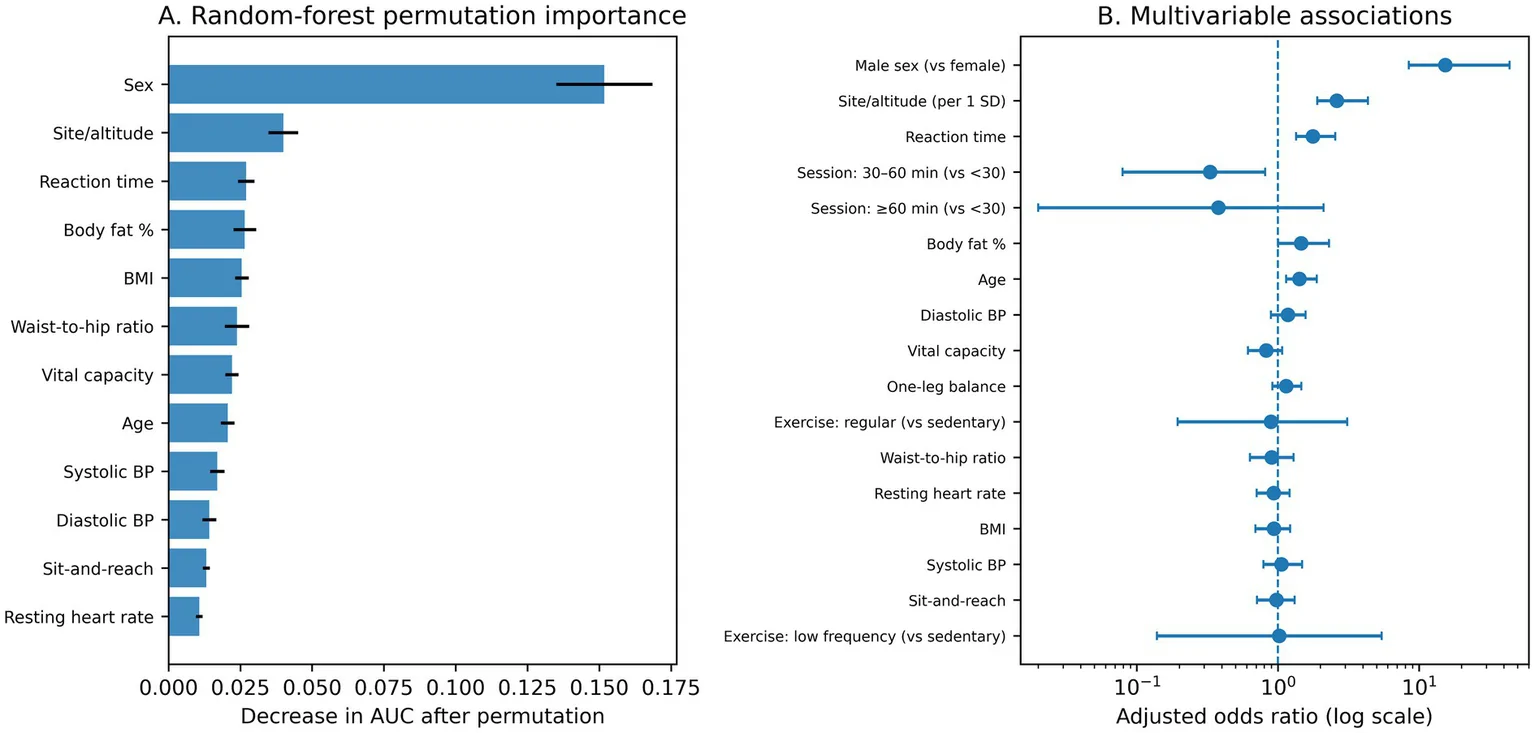
Variable contributions and descriptive adjusted associations. **(A)** Random-forest permutation importance, expressed as decrease in AUC after permutation; bars show mean and standard deviation across permutations. **(B)** Adjusted odds ratios (95% CI) from a multivariable logistic model; continuous variables are per 1-SD increase. The vertical dashed line marks an odds ratio of 1. These analyses describe the development cohort and do not establish causation.

### Partial dependence

Random-forest partial-dependence plots showed higher predicted probability across the three ordered site/altitude values and with slower reaction time, together with non-linear patterns for recorded body fat percentage and body mass index (Figure 4). These curves condition on the fitted model, can be distorted by correlated predictors, sparse ranges, and measurement error, and should be interpreted only as exploratory descriptions rather than biological dose–response relationships.

**Figure 4:**
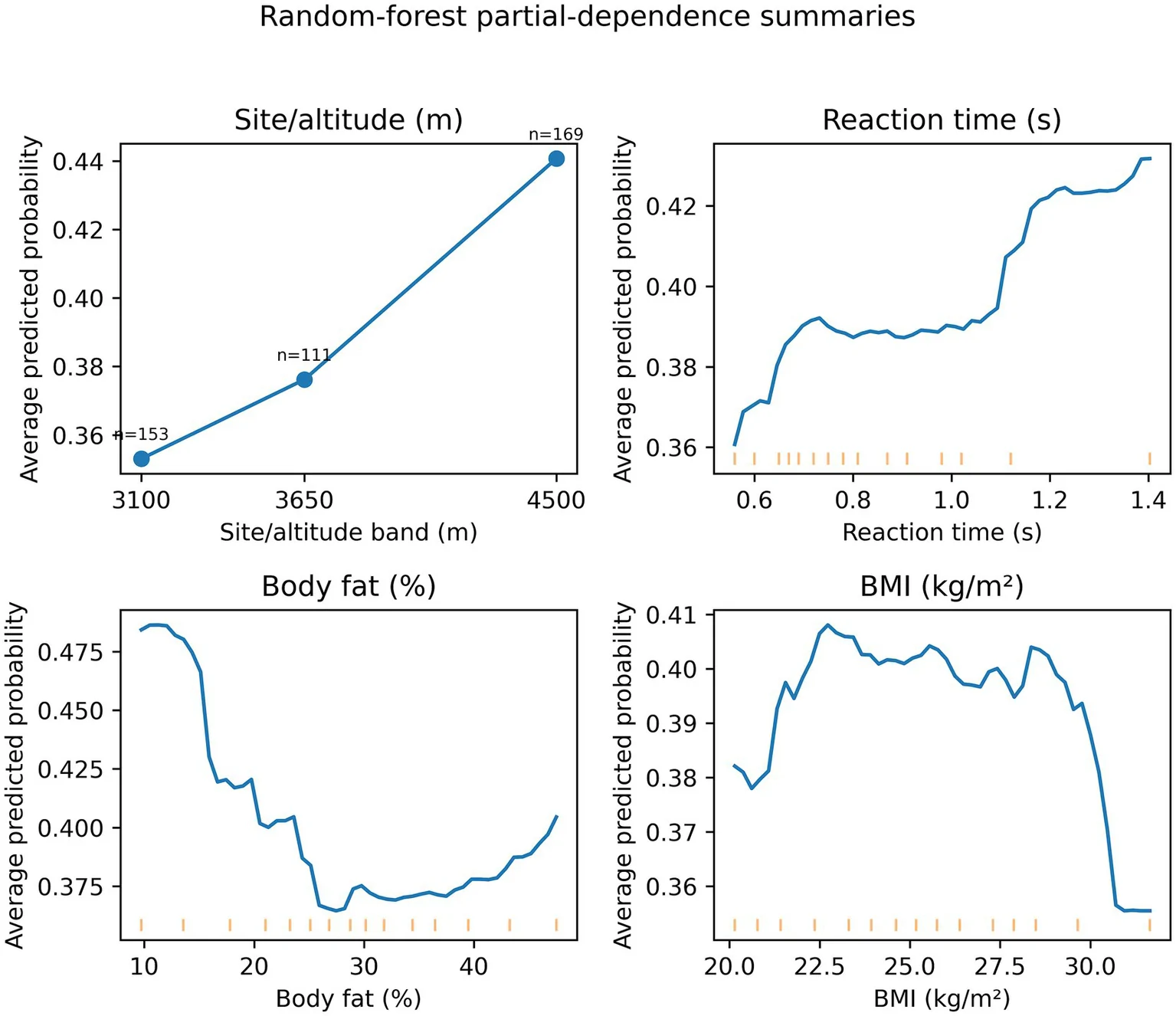
Random-forest partial-dependence summaries for the four leading continuous predictors, showing model-based average predicted probability. Site/altitude is displayed at the three observed bands; rug marks indicate the distributions of continuous predictors. Patterns are exploratory and should not be interpreted as causal dose–response relationships.

### Sensitivity and subgroup analyses

Sensitivity analyses were broadly consistent when blood-pressure predictors were excluded (AUC 0.786), site/altitude was entered categorically (0.777), sedentary participants with a longer recorded session-duration category were reassigned to <30 min (0.777), body fat percentage was excluded (0.785), EWGSOP2 thresholds were used (0.821), or complete cases were analyzed (0.806; Supplementary Table S6). In contrast, AUC fell to 0.653 under the within-sex lowest-tertile outcome, reinforcing that the absolute sex-specific thresholds contributed substantially to the primary discrimination. Candidate-model discrimination was 0.768 in men and 0.632 in women and ranged from 0.698 to 0.847 within site/altitude strata; calibration intervals were wide, particularly for women and the smaller sites (Supplementary Table S7; Supplementary Figure S4).

## Discussion

### Principal findings

The analysis yielded four principal findings. First, all three model families showed moderate discrimination, with no clear advantage of a tree ensemble over penalized logistic regression. Second, the fully specified elastic-net candidate was well calibrated under internal resampling, but its incremental discrimination beyond sex, age and site/altitude was small. Third, performance declined materially when the structural contribution of sex-specific thresholds was reduced. Fourth, leave-one-site/altitude-band-out validation showed lower and heterogeneous performance with calibration drift. Together, these results support a development-stage signal, not a transportable or implementation-ready screening tool.

### The dominant role of sex and the diagnostic threshold

Sex was the dominant predictor because AWGS applies different absolute grip thresholds to men and women (9). A sex-only model already achieved an AUC of 0.660, and adding age and site/altitude increased this to 0.765; the remaining fitness-monitoring variables increased AUC by only 0.016, with a confidence interval that included no increment. Excluding sex reduced AUC to 0.705, and the within-sex tertile definition reduced it to 0.653. Separate sex-specific models were imprecise, especially among women, who contributed only 47 events. The candidate therefore predicts classification under a particular sex-specific definition; it should not be interpreted as a sex-neutral measure of intrinsic muscle biology or as evidence that male sex causes low strength.

### Altitude, hypoxia and muscle strength

The ordered site/altitude band contributed to prediction, and low-strength prevalence was highest at the 4,500-m site. Biological plausibility exists: chronic hypoxia can alter mitochondrial metabolism, oxidative balance and muscle protein turnover, and prior Tibetan work has reported site-related differences in muscle phenotypes and grip reference values (5, 14–16). Nevertheless, one site represented each altitude, so site, altitude, local diet, occupation, physical activity, healthcare access and socioeconomic context could not be separated. The leave-one-site-out results—AUCs of 0.626, 0.695 and 0.759, with calibration intercepts ranging from −0.57 to 0.42—demonstrate heterogeneity rather than an independent altitude effect. All altitude-related findings should therefore be read as site/altitude-associated signals.

### Body composition, reaction time and the wider phenotype

Body composition, reaction time and other functional measures contributed information beyond the simplest benchmarks, but their role is primarily predictive. Both low and high body-composition values may coexist with impaired function, and slower reaction time may capture neuromotor or cognitive correlates of aging. Because these variables were measured concurrently with grip strength, reverse directionality and shared measurement context are unavoidable. Their inclusion is justified only as low-burden markers already present in monitoring records, not as modifiable causes identified by this analysis.

### Comparison with prior work and choice of model

Random forest had the numerically highest AUC, but its confidence interval overlapped those of the other models and its calibration slope departed from 1. The elastic-net model was therefore selected as the sole candidate for future validation because it had similar discrimination, near-ideal internal calibration, a smaller risk of unstable non-linear extrapolation and a fully reproducible equation. This choice is consistent with evidence that machine-learning methods do not reliably outperform well-specified regression in modest clinical datasets (32). The leave-one-site-out calibration shifts indicate that at least intercept recalibration, and possibly broader model updating, may be required in a new setting; such updating must be evaluated in data separate from the development sample (30, 31).

### Public-health implications

The most plausible public-health role is opportunistic triage within an existing monitoring system when grip strength is missing, invalid or unavailable. A positive model flag would trigger standardized grip dynamometry, not a sarcopenia diagnosis. If low strength were confirmed, the next step would be comprehensive assessment of muscle mass and physical performance, review of nutrition, comorbidity and medication, and referral to appropriate resistance or multimodal exercise services (9, 33). Because confirmatory grip testing is low burden, exploratory threshold probabilities of 0.20–0.40 prioritize sensitivity while limiting unnecessary assessments. At a threshold of 0.30, the candidate model had sensitivity 0.784, specificity 0.595, positive predictive value 0.558 and negative predictive value 0.808. A false positive would mainly incur extra assessment time and possible anxiety; a false negative could delay recognition and intervention. Decision-curve net benefit was favorable in the internal out-of-fold data over the exploratory range, but this cannot establish real-world utility. Prospective workflow, safety, equity and impact evaluation would be required before deployment (29, 34).

### Strengths

Strengths include explicit separation of concurrent prediction from causal inference; repeated nested cross-validation with participant-level out-of-fold predictions; confidence intervals for discrimination, calibration and overall accuracy; simple benchmark models that quantify incremental value; a sex-excluded analysis, sex-stratified analyses and definition-based sensitivity analyses; leave-one-site/altitude-band-out internal–external validation; and complete specification of a single candidate model. Reporting follows TRIPOD+AI and STROBE, and code plus machine-readable preprocessing and coefficient files are provided.

### Limitations

Several limitations are important. First, the fixed sample was modest (433 participants and 171 events), limiting model complexity and the precision of subgroup and calibration estimates (22, 23). Second, this was a cross-sectional concurrent-prediction study; it cannot establish temporality, prognosis, or causation, and several predictors are functional correlates of grip strength. Third, all participants came from one monitoring cohort and only three sites. Leave-one-site-out analysis is stronger than random resampling alone but is not independent external validation, and it showed substantial discrimination and calibration heterogeneity (30, 31). Fourth, the outcome was grip strength alone and therefore represents low muscle strength based on consensus thresholds, not complete sarcopenia; performance was strongly influenced by the sex-specific cut-offs, and Tibetan-specific reference values may differ (9, 10, 16). Fifth, site and altitude were inseparable, and nutrition, occupation, socioeconomic status, comorbidity, and medication could not be adjusted. The surveillance algorithm allowed physician-diagnosed treated hypertension, but every available participant also met the measured-BP criterion; the cohort was therefore outcome-selected on BP at the visit, and medication class, regimen, adherence, and control status were unavailable. Sixth, although the collection window and core BP and grip protocols were recovered, participant-level dates, site-specific completion dates, exact BP device identifiers and cuff dimensions, individual BP readings, and the make/models of the bioimpedance, spirometry, and functional-test devices were not retained. BP terminal-digit heaping and uncertain body-fat measurement validity may introduce measurement error, although excluding BP or body fat did not materially change discrimination. Seventh, single within-fold median or mode imputation does not represent imputation uncertainty, although the complete-case result was similar. The exercise variables were self-reported, and 21 sedentary records contained a longer session-duration category; recoding these values did not materially change performance. Sampling weights, strata, and primary-sampling-unit identifiers were unavailable, so the analysis does not represent a design-based estimate for the broader surveillance population. Finally, the age range was restricted to 60–69 years and the cohort comprised Tibetan highlanders meeting the monitoring hypertension criterion, so transportability to other ages, ethnic groups, BP definitions, or settings is unknown.

### Future directions

Future work should externally and prospectively validate the frozen elastic-net specification in independent high-altitude and lower-altitude cohorts, with prespecified evaluation of calibration, discrimination, clinical utility, and performance across sex and site groups (30, 31). Validation studies should use auditable device identifiers and cuff sizes, retain all repeated BP and grip readings, standardize bioimpedance pre-test conditions, record medication, comorbidity, nutrition, and socioeconomic factors, compare the model directly with the simple strategy of measuring grip strength, and determine whether recalibration or model updating is required. Only after successful validation should a prospective impact study test whether model-assisted triage improves confirmation, referral, and functional outcomes without inequitable error rates (34).

## Conclusion

Routine fitness-monitoring data showed moderate ability to predict concurrent low muscle strength based on AWGS grip-strength thresholds in hypertensive adults aged 60–69 years at three high-altitude sites. However, sex, age and site/altitude accounted for most of the discrimination, and leave-one-site-out validation revealed performance and calibration heterogeneity. The fully specified elastic-net model is a candidate for external validation only. Direct grip-strength measurement remains the preferred assessment, and no clinical or public-health implementation is justified without independent validation, recalibration where necessary and prospective evaluation of impact.

## Data Availability

The datasets analyzed for this study are not readily available because the de-identified monitoring data are subject to institutional and ethical restrictions. Requests to access the datasets should be directed to the corresponding author, Weibao Liang (2017020800@m.scnu.edu.cn) and will require permission from the data-holding institution and applicable ethics approval.
