# Supplementary material for: Predicting low muscle strength from routine fitness-monitoring data in hypertensive older adults living at high altitude: development and internal validation of interpretable models
Source: Front Public Health. 2026 Sep 15;14:1916266. doi: 10.3389/fpubh.2026.1916266 (PMC13619500; doi:10.3389/fpubh.2026.1916266)
Supplement: Supplementary file 1 [file Table_1.DOCX]

# Supplementary Material 1

This file provides the expanded computational specification, supplementary tables, and supplementary figures supporting the manuscript. Supplementary Material 2 contains executable code and machine-readable candidate-model files.

# Supplementary methods

**S1. Study data, outcome, and predictor domains**

The analytic cohort comprised 433 of 454 eligible monitoring records after excluding 21 records without grip strength. Data came from the Fifth NPFM fieldwork conducted nationally from August through December 2020; Lhasa monitoring formally began on 1 September 2020, while participant-level dates and the Nyingchi/Nagqu completion dates were not retained. The monitoring algorithm classified hypertension from the mean of three seated BP readings (SBP ≥140 mmHg and/or DBP ≥90 mmHg) or physician-diagnosed hypertension with current antihypertensive treatment. Every record in this analytic file also met the measured-BP criterion. Medication class, regimen, adherence, and treatment-control status were unavailable. The primary outcome was low muscle strength based on AWGS 2019 thresholds (men <28 kg; women <18 kg). Candidate variables were selected before model fitting from routine monitoring domains rather than by univariable outcome screening.

Blood pressure was measured on the right upper arm after at least 5 minutes of seated rest with either a calibrated mercury sphygmomanometer or a validated automated upper-arm device. Three readings were obtained 1–2 minutes apart, and their arithmetic mean was recorded as an integer value. The specific device, cuff dimensions, and individual readings were not retained. Grip strength was measured with a WCS-100 digital dynamometer while standing upright with the tested arm naturally hanging; two maximal dominant-hand trials were performed and the higher value was retained.

Body fat percentage was recorded by bioelectrical impedance analysis, but analyzer model and pre-test conditions were unavailable. Vital capacity used a spirometer and the maximum of two complete expiratory trials after maximal inspiration. Sit-and-reach, eyes-closed one-leg standing, and choice reaction time followed the NPFM older-adult standard, with two trials and the best result retained; the reaction-time test comprised five audiovisual signals. Exact device models for these measures were not retained. Physiologically implausible body-fat and waist-to-hip-ratio values were handled as described in S2, and model sensitivity to excluding body fat was evaluated.

Exercise frequency and session duration were separate questionnaire fields. Primary models preserved the recorded categories. Twenty-one participants classified as sedentary had a 30–60- or ≥60-minute session-duration entry; a sensitivity analysis reassigned those duration entries to <30 minutes because session duration is not logically interpretable when exercise frequency is zero.

**S2. Data cleaning and missing values**

Body-fat values below 5% or above 60% and waist-to-hip ratios above 1.30 were set to missing. Missing continuous variables were median-imputed and categorical variables mode-imputed within each training fold. Imputation parameters learned in a training fold were applied unchanged to its validation fold. This prevented information leakage but did not propagate imputation uncertainty; a complete-case sensitivity analysis was therefore conducted.

**S3. Model preprocessing and tuning**

Elastic-net logistic regression used fold-specific median imputation and standardization for continuous predictors and mode imputation plus one-hot encoding for categorical predictors. Random forest and histogram gradient boosting used the same fold-specific imputation and one-hot encoding without standardization. Hyperparameters were selected by inner four-fold stratified cross-validation maximizing AUC. No class weighting or synthetic oversampling was applied because event prevalence was 39.5% and calibrated probabilities were required.

**S4. Primary repeated nested cross-validation**

Primary performance used a five-fold stratified outer loop repeated twice, with a four-fold inner loop for tuning. Each participant received two out-of-fold probabilities, which were averaged before calculating AUC, PR-AUC, Brier score, calibration intercept, calibration slope, and decision-curve net benefit. Participant-level bootstrap resampling generated uncertainty intervals. The no-skill PR-AUC benchmark equaled the event prevalence (0.395).

**S5. Benchmark and sex-focused analyses**

Post hoc benchmark models included sex alone, sex plus age, sex plus site/altitude, and sex plus age plus site/altitude. All used the same outer splits as the primary analysis. Paired participant-level bootstrap resampling quantified differences in AUC and Brier score between each benchmark and the full elastic-net candidate. A full model excluding sex and separate models in men and women were also evaluated.

**S6. Internal–external cross-validation**

For leave-one-site/altitude-band-out internal–external validation, each of the three bands was held out once. The elastic-net pipeline was tuned using only participants from the other two bands and then evaluated without updating in the held-out band. Altitude remained an ordered numeric input known for the held-out setting. This analysis assesses within-cohort geographic transportability but is not independent external validation.

**S7. Decision context, thresholds, and calibration**

The candidate output was framed as an opportunistic triage flag when a valid grip-strength measurement was unavailable or unusable. A positive flag would prompt standardized grip measurement; a confirmed low value would then prompt full sarcopenia assessment rather than direct diagnosis from the model. Thresholds 0.20–0.40 were emphasized because confirmatory grip testing is low burden and missed low-strength cases were considered more consequential than additional assessment. Threshold 0.50 and the Youden-index operating point were also reported. False positives entail extra assessment and possible anxiety; false negatives may delay confirmation and intervention.

**S8. Sensitivity, subgroup, and interpretation analyses**

Sensitivity analyses excluded SBP and DBP, treated site/altitude categorically, reassigned longer session-duration entries among sedentary participants to <30 minutes, excluded body fat percentage, used EWGSOP2 thresholds, used a within-sex lowest-tertile outcome, and restricted analysis to complete cases. Candidate-model performance was summarized within sex and site/altitude strata. Random-forest permutation importance and partial-dependence summaries were descriptive; adjusted odds ratios came from a separate unpenalized multivariable logistic model and were not interpreted causally.

**S9. Frozen candidate-model specification**

Elastic-net logistic regression was selected as the single candidate for future external validation because discrimination was similar across model families, calibration was closest to ideal, and the model can be transported as an explicit equation. The modal tuning configuration across the 10 outer fits (C=0.30; l1 ratio=1.00) was refitted to all 433 participants solely to freeze coefficients and preprocessing constants. Performance estimates were not taken from this full-data refit. For a new record, continuous values are median-imputed, standardized as z=(x−mean)/SD using Table S10A, categorical indicators are coded using Table S10B, and p=1/[1+exp(−η)], where η is the intercept plus the coefficient-weighted predictors in Table S9.

**S10. Reproducibility**

The verified analysis environment was Python 3.13.5 with scikit-learn 1.8.0, NumPy 2.3.5, and SciPy 1.17.0. Random seeds, grids, aggregate outputs, the frozen model object, coefficient and preprocessing files, and an executable analysis script are supplied in Supplementary Material 2. Individual-level data are not included because institutional and ethics restrictions apply.

# Supplementary tables

**Table S1. Candidate predictors, coding, missingness, and model handling.**

| **Predictor** | **Domain** | **Unit/levels** | **Missing, n (%)** | **Model handling** |
| --- | --- | --- | --- | --- |
| Age | Demographic | years | 0 (0.0%) | Continuous; median imputation, then standardization for elastic-net |
| Sex | Demographic | Female/Male | 0 (0.0%) | Categorical; mode imputation; female reference |
| BMI | Body composition | kg/m² | 0 (0.0%) | Continuous; median imputation, then standardization for elastic-net |
| Body fat percentage | Body composition | % | 15 (3.5%) | BIA value; analyzer model/pre-test conditions unavailable. Values <5% or >60% set to missing; sensitivity analysis excluded this predictor. |
| Waist-to-hip ratio | Body composition | ratio | 16 (3.7%) | Values >1.30 set to missing; then continuous processing |
| Resting heart rate | Cardiovascular | beats/min | 0 (0.0%) | Continuous processing |
| Systolic blood pressure | Cardiovascular | mmHg | 0 (0.0%) | Right arm after ≥5-min seated rest; mean of three readings 1–2 min apart. Device/cuff/replicate metadata unavailable; removed with DBP in sensitivity analysis. |
| Diastolic blood pressure | Cardiovascular | mmHg | 0 (0.0%) | Right arm after ≥5-min seated rest; mean of three readings 1–2 min apart. Device/cuff/replicate metadata unavailable; removed with SBP in sensitivity analysis. |
| Vital capacity | Respiratory | mL | 68 (15.7%) | Spirometry; two trials, maximum retained. Exact device model unavailable; continuous processing. |
| Sit-and-reach | Functional/neuromotor | cm | 1 (0.2%) | Two trials, best retained; exact device model unavailable; continuous processing. |
| One-leg standing, eyes closed | Functional/neuromotor | s | 8 (1.8%) | Two trials, best retained; exact timing-device model unavailable; continuous processing. |
| Choice reaction time | Functional/neuromotor | s | 17 (3.9%) | Five audiovisual signals per trial; two trials, best retained; exact device model unavailable; continuous processing. |
| Exercise frequency | Physical activity | Sedentary; low (1–2 times/week); regular (≥3 times/week) | 0 (0.0%) | Categorical; sedentary reference |
| Session duration | Physical activity | <30; 30–60; ≥60 min | 0 (0.0%) | Recorded <30, 30–60, or ≥60 min; sensitivity reassigned longer entries among sedentary participants to <30 min. |
| Site/altitude band | Context | 3100, 3650, 4500 m | 0 (0.0%) | Primary coding continuous; categorical sensitivity analysis |

*All variables were recorded during the monitoring encounter. Site and altitude were perfectly collinear because one site represented each altitude band. Functional variables were retained as concurrent triage markers, not causal antecedents.*

**Table S2. Missing data among the 433 analyzed participants (variables with any missing values).**

| **Variable** | **Missing, n** | **Missing, %** |
| --- | --- | --- |
| Vital capacity | 68 | 15.7 |
| Walking days/week | 48 | 11.1 |
| Walking min/day | 48 | 11.1 |
| Reaction time | 17 | 3.9 |
| Waist-to-hip ratio (after cleaning) | 16 | 3.7 |
| Body fat % (after cleaning) | 15 | 3.5 |
| Hip circumference | 13 | 3.0 |
| One-leg balance | 8 | 1.8 |
| Chest circumference | 5 | 1.2 |
| Waist circumference | 3 | 0.7 |
| Sit-and-reach | 1 | 0.2 |

*Walking variables were descriptive only and were not included in the 15-predictor model. Model predictors were imputed within cross-validation training folds. Body-fat percentage and waist-to-hip ratio are reported after cleaning physiologically implausible values.*

**Table S3. Model families, preprocessing, hyperparameter grids, and selected configurations.**

| **Model** | **Preprocessing/fixed settings** | **Tuned grid** | **Most frequent selected setting** |
| --- | --- | --- | --- |
| Elastic-net logistic | Continuous: median imputation + standardization. Categorical: mode imputation + one-hot encoding; reference levels dropped. | C: 0.01, 0.03, 0.10, 0.30, 1, 3, 10; l1 ratio: 0, 0.25, 0.50, 0.75, 1.00. | C=0.3; l1 ratio=1.0 (5/10 outer fits). Frozen candidate used this modal configuration. |
| Random forest | Median/mode imputation + one-hot encoding; no standardization. n_estimators fixed at 500. | max_depth: 3, 5, 7; min_samples_leaf: 5, 10, 20; max_features: sqrt, 0.75. | depth=7; leaf=5; max_features=sqrt (4/10). |
| Histogram gradient boosting | Median/mode imputation + one-hot encoding; no standardization. max_iter=200; early_stopping=False. | learning_rate: 0.03, 0.07; max_leaf_nodes: 3, 7, 15; min_samples_leaf: 10, 20, 30; l2: 0, 1. | No dominant setting; the two most frequent configurations were each selected in 2/10 outer fits. |

*Inner cross-validation optimized AUC. All preprocessing was fitted using training data only.*

**Table S4. Benchmark, sex-excluded, and sex-stratified model performance.**

| **Model** | **n/events** | **AUC (95% CI)** | **PR-AUC (95% CI)** | **Brier (95% CI)** | **Cal. slope (95% CI)** | **Cal. intercept (95% CI)** | **ΔAUC full−model (95% CI)** |
| --- | --- | --- | --- | --- | --- | --- | --- |
| Full elastic-net candidate | 433/171 | 0.781 (0.732–0.825) | 0.725 (0.656–0.797) | 0.181 (0.162–0.200) | 0.99 (0.78–1.24) | -0.00 (-0.22 to 0.24) | Reference |
| Sex alone | 433/171 | 0.660 (0.605–0.713) | 0.506 (0.439–0.580) | 0.206 (0.189–0.224) | 0.98 (0.73–1.27) | -0.01 (-0.24 to 0.21) | 0.121 (0.076–0.164) |
| Sex + age | 433/171 | 0.737 (0.691–0.781) | 0.612 (0.537–0.695) | 0.200 (0.184–0.218) | 0.98 (0.76–1.23) | -0.01 (-0.23 to 0.22) | 0.044 (0.008–0.077) |
| Sex + site/altitude | 433/171 | 0.732 (0.681–0.783) | 0.650 (0.569–0.733) | 0.194 (0.176–0.212) | 0.96 (0.74–1.24) | -0.01 (-0.24 to 0.20) | 0.049 (0.014–0.083) |
| Sex + age + site/altitude | 433/171 | 0.765 (0.718–0.811) | 0.702 (0.627–0.775) | 0.187 (0.169–0.207) | 0.96 (0.75–1.22) | -0.01 (-0.23 to 0.21) | 0.016 (-0.009–0.041) |
| Overall, sex excluded | 433/171 | 0.705 (0.649–0.754) | 0.634 (0.556–0.709) | 0.210 (0.192–0.228) | 0.81 (0.57–1.08) | -0.06 (-0.28 to 0.14) | — |
| Male | 213/124 | 0.783 (0.721–0.844) | 0.857 (0.801–0.906) | 0.191 (0.163–0.221) | 0.80 (0.56–1.17) | 0.06 (-0.29 to 0.39) | — |
| Female | 220/47 | 0.640 (0.545–0.725) | 0.313 (0.221–0.444) | 0.163 (0.133–0.194) | 0.64 (0.12–1.27) | -0.41 (-1.23 to 0.42) | — |

*Benchmark models were unpenalized logistic regressions evaluated on the same repeated outer splits. ΔAUC is full elastic-net minus the listed benchmark; positive values favor the full model. Sex-stratified rows are separate elastic-net models, not subgroup evaluation of the full candidate.*

**Table S5. Candidate-model operating characteristics at exploratory thresholds.**

| **Operating point** | **Threshold** | **Sensitivity (95% CI)** | **Specificity (95% CI)** | **PPV (95% CI)** | **NPV (95% CI)** | **Accuracy (95% CI)** | **Net benefit (95% CI)** |
| --- | --- | --- | --- | --- | --- | --- | --- |
| Exploratory | 0.200 | 0.901 (0.853–0.944) | 0.382 (0.322–0.441) | 0.487 (0.431–0.541) | 0.855 (0.786–0.917) | 0.587 (0.540–0.633) | 0.262 (0.209 to 0.314) |
| Exploratory | 0.300 | 0.784 (0.722–0.845) | 0.595 (0.537–0.655) | 0.558 (0.496–0.619) | 0.808 (0.753–0.862) | 0.670 (0.626–0.714) | 0.205 (0.153 to 0.256) |
| Exploratory | 0.400 | 0.684 (0.615–0.753) | 0.710 (0.656–0.766) | 0.606 (0.538–0.675) | 0.775 (0.724–0.827) | 0.700 (0.656–0.741) | 0.153 (0.101 to 0.206) |
| Youden J | 0.481 | 0.596 (0.524–0.669) | 0.824 (0.778–0.871) | 0.689 (0.615–0.763) | 0.758 (0.706–0.806) | 0.734 (0.693–0.776) | 0.137 (0.086 to 0.189) |
| 0.50 | 0.500 | 0.556 (0.481–0.631) | 0.844 (0.795–0.886) | 0.699 (0.619–0.775) | 0.744 (0.694–0.792) | 0.730 (0.688–0.771) | 0.125 (0.074 to 0.176) |

*Metrics use averaged participant-level out-of-fold probabilities from repeated nested cross-validation. The Youden threshold was 0.4807 and is displayed as 0.481. Confidence intervals are participant-level bootstrap intervals with each threshold held fixed. These are descriptive operating points, not approved deployment thresholds.*

**Table S6. Sensitivity analyses under alternative feature sets and outcome definitions.**

| **Scenario** | **n** | **Events** | **AUC (95% CI)** | **PR-AUC** | **Brier** |
| --- | --- | --- | --- | --- | --- |
| Primary outcome, all predictors | 433 | 171 | 0.781 (0.730–0.828) | 0.738 | 0.180 |
| Excluding systolic and diastolic BP | 433 | 171 | 0.786 (0.742–0.830) | 0.726 | 0.180 |
| Site/altitude entered as categorical | 433 | 171 | 0.777 (0.728–0.821) | 0.729 | 0.183 |
| EWGSOP2 grip-strength cut-offs | 433 | 137 | 0.821 (0.771–0.866) | 0.756 | 0.148 |
| Sex-specific lowest-tertile outcome | 433 | 145 | 0.653 (0.593–0.707) | 0.515 | 0.207 |
| Complete-case analysis | 325 | 128 | 0.806 (0.755–0.852) | 0.764 | 0.169 |
| Sedentary duration reassigned to <30 min | 433 | 171 | 0.777 (0.732–0.820) | 0.732 | 0.182 |
| Excluding body-fat percentage | 433 | 171 | 0.785 (0.739–0.830) | 0.750 | 0.179 |

*Sensitivity analyses used the fixed candidate configuration (C=0.30; l1 ratio=1.00) under repeated stratified five-fold cross-validation. Thus, the primary fixed-configuration AUC differs slightly from the nested-cross-validation estimate in Table 2 of the manuscript. The duration-recoding and body-fat-exclusion analyses addressed measurement-protocol uncertainties; neither materially altered discrimination.*

**Table S7. Full candidate-model performance within sex and site/altitude strata.**

| **Stratum** | **n** | **Events** | **AUC (95% CI)** | **PR-AUC** | **Brier** | **Cal. slope (95% CI)** | **Cal. intercept (95% CI)** |
| --- | --- | --- | --- | --- | --- | --- | --- |
| Overall | 433 | 171 | 0.781 (0.735–0.823) | 0.725 | 0.181 | 0.99 (0.78–1.23) | -0.00 (-0.21 to 0.23) |
| Male | 213 | 124 | 0.768 (0.709–0.830) | 0.811 | 0.196 | 1.18 (0.83–1.68) | 0.02 (-0.33 to 0.32) |
| Female | 220 | 47 | 0.632 (0.545–0.714) | 0.305 | 0.166 | 0.57 (0.19–1.00) | -0.56 (-1.16 to 0.05) |
| 3100 m | 153 | 46 | 0.698 (0.612–0.781) | 0.512 | 0.192 | 0.67 (0.40–1.01) | -0.25 (-0.71 to 0.19) |
| 3650 m | 111 | 40 | 0.713 (0.607–0.813) | 0.613 | 0.199 | 0.81 (0.37–1.42) | -0.12 (-0.60 to 0.32) |
| 4500 m | 169 | 85 | 0.847 (0.780–0.907) | 0.833 | 0.158 | 1.39 (1.01–1.94) | 0.08 (-0.28 to 0.46) |

*These rows evaluate the full candidate’s out-of-fold predictions within each stratum. They differ from the separately refitted male and female models in Table S4. Intervals are wide because subgroup sample sizes and event counts are limited.*

**Table S8. Leave-one-site/altitude-band-out internal–external validation.**

**Table S8A. Discrimination and overall accuracy.**

| **Held-out band** | **n** | **Events** | **Observed prevalence** | **Mean predicted** | **AUC (95% CI)** | **PR-AUC (95% CI)** | **Brier (95% CI)** |
| --- | --- | --- | --- | --- | --- | --- | --- |
| 3100 m | 153 | 46 | 0.301 | 0.330 | 0.626 (0.535–0.716) | 0.406 (0.303–0.544) | 0.238 (0.198–0.280) |
| 3650 m | 111 | 40 | 0.360 | 0.391 | 0.695 (0.583–0.800) | 0.602 (0.456–0.736) | 0.208 (0.174–0.245) |
| 4500 m | 169 | 85 | 0.503 | 0.426 | 0.759 (0.683–0.829) | 0.793 (0.701–0.871) | 0.206 (0.180–0.234) |
| Pooled held-out | 433 | 171 | 0.395 | — | 0.695 (0.644–0.746) | 0.578 (0.506–0.659) | 0.218 (0.197–0.238) |

**Table S8B. Calibration and selected tuning parameters.**

| **Held-out band** | **Calibration slope (95% CI)** | **Calibration intercept (95% CI)** | **Parameters selected in the two-site training set** |
| --- | --- | --- | --- |
| 3100 m | 0.31 (0.10–0.54) | -0.57 (-0.98 to -0.18) | C=10; l1 ratio=0.75 |
| 3650 m | 0.74 (0.31–1.35) | -0.24 (-0.70 to 0.18) | C=0.3; l1 ratio=1.0 |
| 4500 m | 1.15 (0.75–1.71) | 0.42 (0.07 to 0.77) | C=1; l1 ratio=0.75 |
| Pooled held-out | 0.62 (0.44–0.83) | -0.09 (-0.31 to 0.12) | Not applicable |

*One site represented each altitude band. The held-out analysis therefore estimates combined site/altitude transportability and cannot isolate altitude from other site-level differences.*

**Table S9. Frozen elastic-net candidate equation: intercept and coefficients.**

| **Term** | **Coding** | **Coefficient** |
| --- | --- | --- |
| Intercept | — | -1.512079 |
| Age | Standardized continuous value | 0.306316 |
| BMI | Standardized continuous value | -0.014163 |
| Body fat % | Standardized continuous value | 0.126152 |
| Waist-to-hip ratio | Standardized continuous value | 0.000000 |
| Resting heart rate | Standardized continuous value | 0.000000 |
| Systolic BP | Standardized continuous value | 0.060986 |
| Diastolic BP | Standardized continuous value | 0.110627 |
| Vital capacity | Standardized continuous value | -0.146623 |
| Sit-and-reach | Standardized continuous value | 0.000000 |
| One-leg balance | Standardized continuous value | 0.075612 |
| Reaction time | Standardized continuous value | 0.483624 |
| Site/altitude (per 1 SD) | Standardized continuous value | 0.708623 |
| Male sex (vs female) | Indicator (1=yes, 0=reference) | 2.073417 |
| Exercise: low frequency (vs sedentary) | Indicator (1=yes, 0=reference) | 0.000000 |
| Exercise: regular (vs sedentary) | Indicator (1=yes, 0=reference) | 0.000000 |
| Session: 30–60 min (vs <30) | Indicator (1=yes, 0=reference) | -0.493778 |
| Session: ≥60 min (vs <30) | Indicator (1=yes, 0=reference) | 0.000000 |

*The linear predictor is η=intercept+ΣβjXj, and probability p=1/[1+exp(−η)]. Zero coefficients reflect shrinkage by the lasso-dominant modal elastic-net configuration. This full-data refit freezes the model for validation; it was not used to estimate performance.*

**Table S10. Frozen preprocessing constants and categorical coding.**

**Table S10A. Numeric-variable imputation and standardization constants.**

| **Variable** | **Median for imputation** | **Mean after imputation** | **SD after imputation** |
| --- | --- | --- | --- |
| Age | 64 | 64.358 | 2.81513 |
| BMI | 25.17 | 25.3355 | 3.66291 |
| Body fat % | 28.74 | 28.7899 | 11.2697 |
| Waist-to-hip ratio | 0.94 | 0.941778 | 0.0732286 |
| Resting heart rate | 79 | 78.8222 | 8.99824 |
| Systolic BP | 140 | 137.076 | 11.5131 |
| Diastolic BP | 98 | 96.3626 | 8.66013 |
| Vital capacity | 1560 | 1614.86 | 448.018 |
| Sit-and-reach | 3.3 | 2.53418 | 6.98031 |
| One-leg balance | 6 | 8.15242 | 8.90726 |
| Reaction time | 0.78 | 0.84358 | 0.261405 |
| Site/altitude (per 1 SD) | 3650 | 3787.41 | 608.274 |

*After median imputation, standardized values are z=(x−mean)/SD. Means and SDs were calculated in the full 433-participant refit solely for the frozen model specification.*

**Table S10B. Categorical order, reference levels, and imputation modes.**

| **Variable** | **Category order** | **Reference level** | **Mode for imputation** |
| --- | --- | --- | --- |
| Sex | Female; Male | Female | Female |
| Exercise Freq | Sedentary; Low frequency; Regular | Sedentary | Sedentary |
| Session Duration | <30 min; 30-60 min; >=60 min | <30 min | <30 min |

**Table S11. Software, random seeds, and Supplementary Material 2 contents.**

| **Component/file** | **Version/setting** | **Role** |
| --- | --- | --- |
| Python | 3.13.5 | Analysis language |
| scikit-learn | 1.8.0 | Modeling and performance estimation |
| NumPy | 2.3.5 | Array calculations |
| SciPy | 1.17.0 | Statistical calculations |
| Global random seed | 20260730 | Outer splits, model fitting, and deterministic resampling base |
| Outer validation | 5 folds × 2 repeats | Repeated stratified nested cross-validation |
| Inner tuning | 4 stratified folds | AUC-optimized grid search |
| Primary uncertainty | 2000 participant bootstrap samples | AUC, PR-AUC, Brier, calibration slope/intercept |
| Threshold uncertainty | 4000 participant bootstrap samples | Fixed-threshold operating characteristics |
| analysis_pipeline.py | Included in Supplementary Material 2 | Executable analysis workflow; requires the restricted analytic data |
| elastic_net_candidate_model.joblib | Included | Frozen pipeline and coefficients for external validation |
| elastic_net_candidate_model_specification.json | Included | Machine-readable model, coding, and preprocessing specification |
| CSV aggregate outputs | Included | Performance, thresholds, coefficients, preprocessing, IECV, sensitivity, and subgroup results |

*The supplied package contains no participant-level data.*

# Supplementary figures

**Figure S1. Distribution of recorded grip strength by sex with AWGS 2019 sex-specific thresholds (men <28 kg; women <18 kg).** The thresholds classified a larger proportion of men than women as having low muscle strength in this cohort.

**Figure S2. Spearman correlation matrix for continuous monitoring variables.** Grip strength is shown only as a descriptive reference and was not used as a predictor. Site/altitude is an ordered site-level variable, not an independently identified environmental exposure.

**Figure S3. Precision–recall curves for the three model families.** The horizontal no-skill line equals event prevalence (0.395).

**Figure S4. Full elastic-net candidate-model AUC within sex and site/altitude strata.** Wide intervals reflect limited subgroup sample sizes and events.

**Figure S5. AUC for simple benchmark models, the sex-excluded model, and separately refitted sex-stratified models. The vertical dotted line marks the full elastic-net candidate AUC.**

**Figure S6. Leave-one-site/altitude-band-out internal–external validation of the elastic-net candidate.** One site represented each altitude band; the figure assesses combined site/altitude transportability.
